# Surgical Management of Periapical Lesion with Dens in Dente

**DOI:** 10.5005/jp-journals-10005-1040

**Published:** 2009-04-26

**Authors:** MK Jindal, Md Asadullah, SK Misra

**Affiliations:** 1 Reader and Chairman, Department of Pedodontics, ZA Dental College, AMU, Aligarh, Uttar Pradesh, India; 2 Tutor, Department of Oral Pathology and Medicine, ZA Dental College, AMU, Aligarh, Uttar Pradesh, India; 3 Senior Lecturer, Department of Conservative Dentistry, ZA Dental College, AMU, Aligarh, Uttar Pradesh, India

**Keywords:** Dens invaginatus, complex internal anatomy, root canal, apicoectomy, in folding.

## Abstract

The management of one case of dens in Dente (Dens invaginatus) in maxillary lateral incisor with history of trauma to maxillary central incisor with periradicular lesion is reported. The patient presented with pain and fracture of anterior tooth. Despite of complex anatomy and diagnosis of dens invaginatus, surgical root canal (Apicoectomy) was performed successfully. Further more essential clinical considerations and treatment options are suggested. Early diagnosis and management are important to avoid complications.

## INTRODUCTION

Dens in dente is an anomaly of development resulting from deepening or invagination of the enamel organ into the dental papilla which begins at the crown and often extends to the root, before calcification of the dental tissues. Affected tooth shows a deep in folding of enamel and dentin starting from the foramen coecum or even the tip of the cusps and which may extend deep into the root. Tooth most affected is maxillary lateral incisors and bilateral occurrence is not uncommon. The malformation showed a broad spectrum of morphologic variations and frequently results in early pulp necrosis. Root canal therapy may present severe problems because of complex anatomy of the tooth. Bilateral occurrences are 43% of all cases. There is no sex predilection, exhibits a high degree of inheritability.

The presumed etiology has been related either to focal growth retardation/or focal growth stimulation or to localized external pressure to certain areas of the tooth bud.

Öhlers is most popular system used to classify Dens Invaginatus as follows:

**Type 1:** Cases are those in which invaginations end as a blind sec within the crown.

**Type 2:** The invagination extends epically beyond the cement enamel junction.

**Type 3:** The invagination extends beyond the cement enamel junction and a second "apical foramen" is evident.

Radiographically this anomaly demonstrates a radioopaque invagination equal in density to enamel extending from cingulum into the root canal.

Various techniques of treating dens invaginatus have been reported including conservative restorative treatment, non-surgical root canal treatment, endodontics surgery, intentional replantation and extraction.

This article presents one case of surgical management of type II dens invaginatus and discusses the various treatment options available in its management.

## CASE REPORT

A female patient of 11 years reported with history of pain, pus discharge and fractured central incisor in the Department of Oral Medicine and Radiology OPD in Dr ZA Dental College and Hospital, AMU Aligarh.

**Fig. 1.**
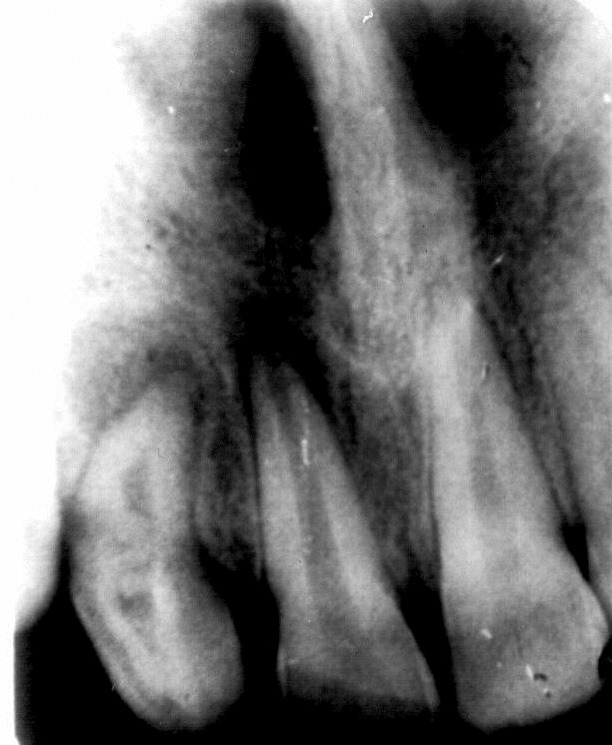
Preoperative dense in dente with periapical pathology

Periapical radiograph of maxillary anterior teeth revealed that fracture of central incisor with periapical pathology related to both central and lateral incisor. There was an invagination extending beyond the CEJ toward apex of lateral incisor (Fig. 1).

The patient is referred to Pedodontics Department for further management.

**Fig. 2.**
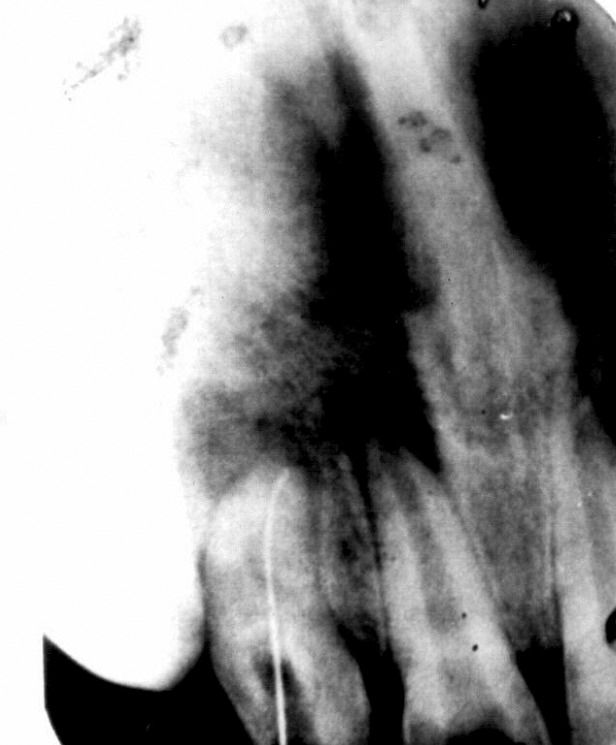
File in canal

**Fig. 3.**
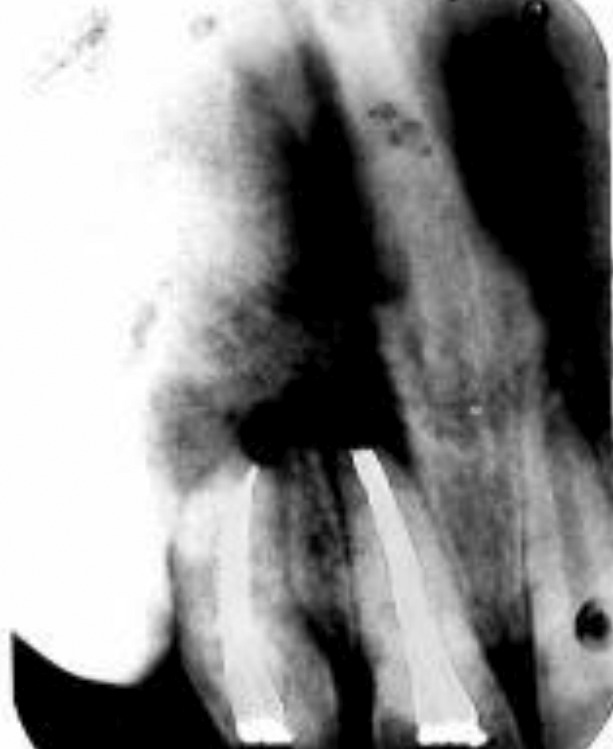
Postobturation

On examination it was found that apical closure of maxillary central incisors has not taken place and persistent sinus was present. Hence, periapical surgery was planned. After anesthetizing the tooth, the pulp chamber was opened as there is big invaginatus space involving cementoenamel junction (Type II), no rudimentary canal was found. During biomechanical canal preparation invaginatus space is perforated by using long tapering bur. Once the K-file went upto apex then further enlargement was carried out using Haedstroem file (Fig. 2). After removing the complete obstruction from lateral incisor, little amount of root apex of both the lateral and central incisor was removed. Then perapical pathologic tissue was removed and defect was irrigated with normal saline followed by complete obturation of canal as well as of invaginatus space with the help of Gutta Percha and Root Canal Sealer (Fig. 3).

## DISCUSSION

An early diagnosis of dens in dente is crucial and require thorough clinical examination of all teeth. This invagination acts as niche for bacterial growth and may jeopardize the status of main canal and most of the time root canal treatment becomes difficult. An early detection and sealing of its opening with restorative materials can effectively prevent those complications. Another treatment modality is treating the invaginatus yet retaining pulp vitality in the separated part. There is postoperative sensitivity as well as it may undergo inflammation and necrosis due to impact of irrigation solutions and sealers.

The merging of main canal with the invaginatus space (As done in this case) is alternative treatment to facilitate the proper biomechanical preparation. However, it may lead to increased risk of root fracture because of the thin walls and the loss of tooth materials.

Presence of invaginatus always creates present technical difficulties with respect to their management because of complicated canal morphology.

## CONCLUSION

Patients generally do not report for the treatment of dens invaginatus as it does not present with any clinical symptoms. It is diagnosed either during routine dental examination or investigating for other complains. As in this case patient came for treatment of fractured maxillary central incisor which was bothering him. While investigating for central incisor, a dense invaginatus in lateral incisor is noticed with large periapical pathology. That is why clinician must examine every patient thoroughly so that dental anomalies may be detected early and properly treated.
